# Complete chloroplast genome of a widely distributed species in southwest China, *Hemiphragma heterophyllum* Wall. (Scrophulariaceae)

**DOI:** 10.1080/23802359.2019.1676678

**Published:** 2019-10-11

**Authors:** Xiaobo Wu, Dequan Zhang

**Affiliations:** a College of Pharmacy and Chemistry, Dali University, Dali, China;; b Institute of Materia Medica, Dali University, Dali, China;; c Key Laboratory of Yunnan Provincial Higher Education Institutions for Development of Yunnan Daodi Medicinal Materials Resources, Dali, China

**Keywords:** *Hemiphragma heterophyllum*, widely distributed species, complete chloroplast genome, phylogenetic analysis

## Abstract

*Hemiphragma heterophyllum* Wall., a widely distributed species in southwest China, belongs to a monotypic genus, *Hemiphragma* Wall. in the Scrophulariaceae. To date, there has been no study on its complete chloroplast genome. Hence, we reported the first complete chloroplast genome sequence of *H*. *heterophyllum* here. The genome sequences of two individuals were extremely similar except slight differences on sequence lengths (152,707 bp and 152,700 bp, respectively). There was a typical circular quadripartite structure. A total of 113 genes were annotated, including 79 protein-coding genes, 29 tRNA genes, four rRNA genes, and one pseudogene. Among the annotated genes, 16 genes contained one intron, whereas another two genes (ycf3 and clpP) possessed two gene introns. Total GC content is 38.1% in the chloroplast genome. Moreover, a total of 63 simple sequence repeats (SSRs) with five types were detected. Phylogenetic analysis revealed that *Hemiphragma* was closely related to *Veronica* L. and *Veronicastrum* Heist. *ex* Farbic.

*Hemiphragma heterophyllum* Wall. is a perennial herb belonging to the genus *Hemiphragma* Wall. in the family Scrophulariaceae and there is only one species in this genus (Hong et al. 1998). The species is widely distributed in Yunnan, Tibet, Sichuan, Guizhou, and adjacent regions in China, and also it could be found in Nepal, India, and the Philippines. It usually grows in alpine grassland or rock crevices with an altitude of 3000–4000 m (Hong et al. 1998). The whole plant of *H. heterophyllum* can be used for promoting blood circulation, heat-clearing and detoxification, as well as eliminating wind and dampness (Long and Li 2004; He et al. 2013). Therefore, it is listed as folk medicine by Bai and Dai Nationalities in western Yunnan, China (Lee et al. 2008). However, the current researches on this species mainly focussed on its chemical compositions, but rarely involved its molecular biology (Tian and Zhou 2004; Yang et al. 2004; Dai et al. 2019; Lu et al. 2019). Therefore, this study reported complete chloroplast (cp) genome of *H. heterophyllum* and revealed its phylogenetic relationships with closely related species or genera in the Scrophulariaceae.

In this study, fresh and healthy leaves of two individuals were collected from Cangshan Mountains, Dali, Yunnan province, China (N25°53′29.13″, E100°02′32.61″) and used as molecular materials. Meanwhile, 3–5 voucher specimens (No. ZDQ17020) with fruits were collected at the same time and deposited at the Herbarium of Medicinal Plants and Crude Drugs of the College of Pharmacy and Chemistry, Dali University. Total genomic DNA was extracted from dried leaf using a modified CTAB method (Doyle and Doyle 1987) and sequenced using the Illumina Hiseq 2500 platform (Novogene, Tianjing, China) with pair-end (2 × 300 bp) library. Raw data were filtered using Trimmomatic v.0.32 software with default parameters (Bolger et al. 2014). Subsequently, the trimmed reads were assembled into contigs utilising GetOrganelle.py (Jin et al. 2018) with *Veronicastrum sibiricum* (L.) Pennell (NC_031345) as reference. Finally, complete chloroplast genome sequences of *H. heterophyllum* were annotated in Geneious 8.0.2 (Kearse et al. 2012) and then submitted to GenBank (accession numbers: MN383191 and MN383192). Moreover, distributions of the simple sequence repeats (SSRs) were explored using the microsatellite search tool MISA (Thiel et al. 2003). To explore phylogenetic position of *H. heterophyllum*, the cpDNA genome sequences of this species were aligned with another 26 reported sequences in the family Scrophulariaceae using MAFFT V.7.1 (Katoh and Standley 2013). Neighbor-joining (NJ) tree was constructed using MEGA 7.0 (Kumar et al. 2016) with 1000 bootstrap values and Kimura 2-parameter model (K2P), and two species (*Scutellaria insignis* Nakai and *S. lateriflora* L.) from the family Labiatae were selected as outgroup taxa.

The annotated results showed that complete chloroplast genome sequences of the two individuals were extremely similar except for slight difference on sequence lengths. Herein, one of the sequences (MN383192) was introduced as the followings. The whole cp genome sequence was 152,707 bp in length with a typical circular quadripartite structure, consisting of a pair of inverted repeat regions (IRa and IRb) of 25,808 bp, which were separated by a large single copy (LSC) region of 83,268 bp and a small single copy (SSC) region of 17,823 bp. A total of 113 genes were annotated in the *H. heterophyllum* cp genome, including 79 protein-coding genes, 29 tRNA genes, four rRNA genes, and one gene was inferred to be pseudogene. Among the annotated genes, 16 genes (*atpF, ndhA, ndhB, petB, petD, rpl2, rpl16, rpoC1, rps12, rps16, trnA-UGC, trnG-UCC, trnI-CAU, trnK-UUU, trnL-UAA,* and *trnV-UAC*) contained one intron, whereas another two genes (*ycf3* and *clpP*) possessed two introns. Total GC content was 38.1%, and the corresponding values in LSC, SSC, and IR regions are 36.2%, 32.1%, and 43.2%, respectively. Furthermore, a total of 63 simple sequence repeats (SSRs) with five types were detected in the *H. heterophyllum* cp genome, including 42 mononucleotide repeats, eight dinucleotide repeats and 13 other types of SSR loci.

The phylogenetic tree showed that two individuals of *H. heterophyllum* were clustered together with *Veronica nakaiana*, *Veronica persica* and *Veronicastrum sibiricum*. The results revealed that the genus *Hemiphragma* was closely related to *Veronica* and *Veronicastrum*. Compared with *Veronica*, the genus was closer to *Veronicastrum* in molecular phylogenetics (Figure 1). On the contrary, the monotypic genus was obviously more distant to *Brandisia*, *Lindenbergia* and *Pedicularis* than other genus. Consequently, this study accurately revealed phylogenetic position of *H. heterophyllum* in the Scrophulariaceae, which would be beneficial to further phylogenetic studies on the related species or genera in the family.

**Figure 1.**
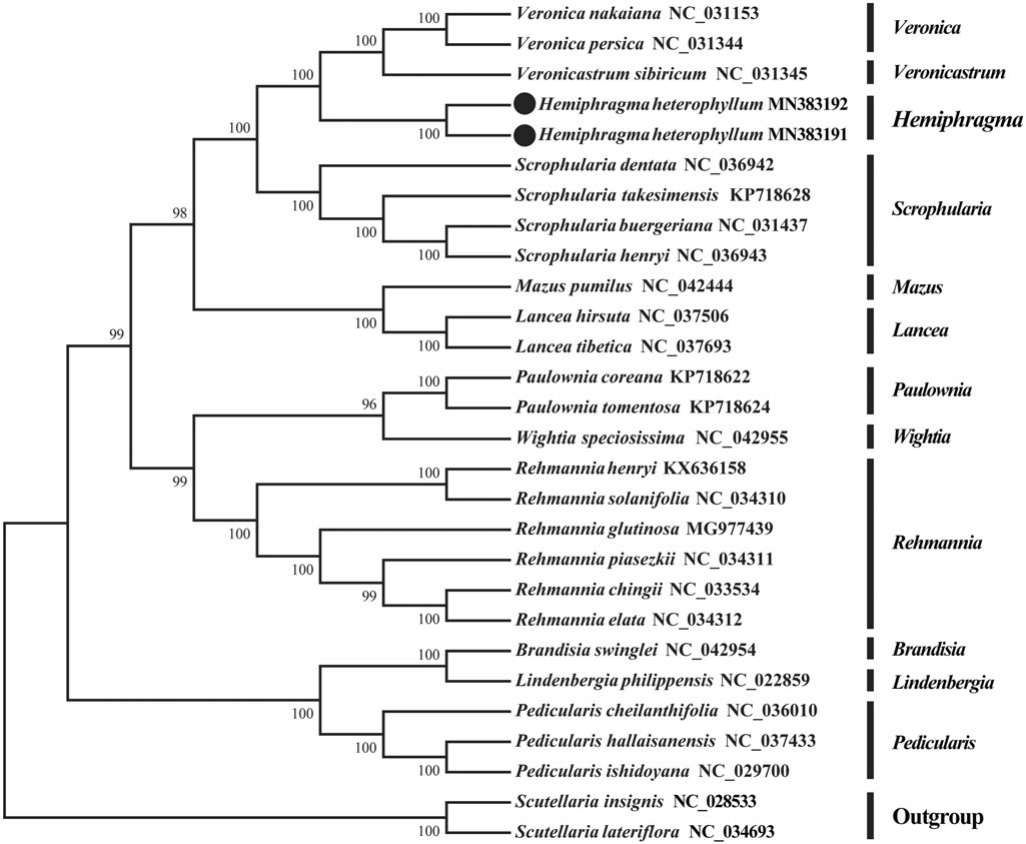
Neighbor-joining (NJ) tree of *H. heterophyllum* and 24 other related species from the family Scrophulariaceae. Numbers above each node are bootstrap support values. *Scutellaria insignis* and *S. lateriflora* from Labiatae were selected as outgroup.
